# How Biodiversity, Climate and Landscape Drive Functional Redundancy of British Butterflies

**DOI:** 10.3390/insects14090722

**Published:** 2023-08-23

**Authors:** Maria Lazarina, Danai-Eleni Michailidou, Mariana Tsianou, Athanasios S. Kallimanis

**Affiliations:** Department of Ecology, Aristotle University of Thessaloniki, 54124 Thessaloniki, Greece; dmichail@bio.auth.gr (D.-E.M.); kalliman@bio.auth.gr (A.S.K.)

**Keywords:** functional diversity, taxonomic diversity, species richness, functional richness, redundancy, β-diversity, abiotic drivers, climate warming

## Abstract

**Simple Summary:**

Functional redundancy, the coexistence of species with similar functional roles, is of critical importance for ecosystem function stability. However, drivers of functional redundancy remain relatively poorly understood. Here, we analyzed four traits of British butterflies and aimed to identify the biotic and abiotic factors that affect the spatial patterns of functional redundancy. The most important factor was taxonomic diversity, with species-rich communities having the highest level of functional redundancy. Temperature was associated with redundancy and with different facets of taxonomic and functional diversity. However, although warmer areas hosted species-rich communities, redundancy was greatest in areas of intermediate mean annual temperature and declined at higher temperatures. This might imply that despite the positive effect of increased temperature on butterfly diversity, warmer and species-rich areas are vulnerable, perhaps due to the land uses dominating in these regions. Landscape heterogeneity promoted species richness, functional redundancy and variation in species and functional composition of the butterfly assemblages, underscoring the importance of maintaining diverse landscapes.

**Abstract:**

Biodiversity promotes the functioning of ecosystems, and functional redundancy safeguards this functioning against environmental changes. However, what drives functional redundancy remains unclear. We analyzed taxonomic diversity, functional diversity (richness and β-diversity) and functional redundancy patterns of British butterflies. We explored the effect of temperature and landscape-related variables on richness and redundancy using generalized additive models, and on β-diversity using generalized dissimilarity models. The species richness-functional richness relationship was saturating, indicating functional redundancy in species-rich communities. Assemblages did not deviate from random expectations regarding functional richness. Temperature exerted a significant effect on all diversity aspects and on redundancy, with the latter relationship being unimodal. Landscape-related variables played a role in driving observed patterns. Although taxonomic and functional β-diversity were highly congruent, the model of taxonomic β-diversity explained more deviance than the model of functional β-diversity did. Species-rich butterfly assemblages exhibited functional redundancy. Climate- and landscape-related variables emerged as significant drivers of diversity and redundancy. Τaxonomic β-diversity was more strongly associated with the environmental gradient, while functional β-diversity was driven more strongly by stochasticity. Temperature promoted species richness and β-diversity, but warmer areas exhibited lower levels of functional redundancy. This might be related to the land uses prevailing in warmer areas (e.g., agricultural intensification).

## 1. Introduction

Through their activities such as land-use change, habitat degradation and resource overexploitation, humans have transformed the biosphere, with scientists warning that we are on the verge of crossing, or we have already crossed, the planetary boundaries [1]. Our planet has drastically changed, with for example frequent extreme weather events, drought, and severe floods and the global warming that is felt almost daily as in the last 100 years, average temperature increased by 0.7 °C, and further increase is expected in the foreseeable future [2,3]. The biodiversity loss is unprecedented and evidence suggests that ecosystem functioning and delivery of ecosystem services is destabilized [4]. Therefore, the urgency to comprehend diversity patterns and underlying processes driving them is crucial to ensuring ecosystem functioning and resilience in order to secure a better future [4,5]. 

Taxonomic diversity is the most widely studied facet of diversity and has been extensively used to explore the biodiversity-ecosystem functioning relationship [6,7]. However, consequences of biodiversity loss on ecosystems depend on facets of biodiversity other than merely species richness, such as functional diversity [8]. Functional diversity, i.e., the diversity of behavioral, physiological and morphological traits within a community, reflects the role of species within ecosystems and their responses to environmental changes [9]. Thus, functional diversity metrics are more strongly associated with ecosystem functioning than taxonomic diversity [6] and can provide unique insights into biodiversity responses to changes and subsequent impacts on ecosystem functioning [10]. However, functional diversity patterns at large spatial scales (but see [11,12,13,14]) and the environmental drivers (e.g., climate, landscape factors) shaping them are not fully elucidated [15].

Biodiversity promotes ecosystem functioning and safeguards it through insurance effects against environmental changes and disturbances. In this context, species-richer communities have more species that perform similar functional roles, ensuring that loss of ecosystem functions by biotic erosion is mitigated [16]. This concept is defined as functional redundancy. Specifically, functional redundancy refers to the presence of multiple distinct species that share functional traits and perform similar, and thus interchangeable, ecosystem functioning in the community. In other words, functional redundancy reflects the functional roles’ overlap within the ecosystem. Consequently, even if the contribution of some species to ecosystem processes decreases or collapses, e.g., in case of abundance decrease or extinction, other species will fill in the “gap”, i.e., compensate for the losses and retain ecosystem functioning levels [17]. Given that species with similar functional roles respond differently to environmental changes, functional redundancy contributes to the temporal stability of ecosystems [18]. 

The coexistence of functionally similar species within a community might signal that abiotic conditions act as environmental filters selecting species with traits adapted to the local conditions [19]. Mapping patterns and drivers of variation in taxonomic and functional β-diversity can shed light on the underlying mechanisms shaping community assembly. Taxonomic β-diversity quantifies the variation in species composition [20], while functional β-diversity is defined as the trait diversity among species from different communities. The simultaneous consideration of taxonomic and functional β-diversity allows us to understand how communities respond to environmental and spatial gradients and disturbances [21]. A considerable amount of knowledge on taxonomic β-diversity across environmental gradients at different spatial and temporal scales has been gained, but the patterns of functional β-diversity (especially at coarse geographic scales and for most taxa) and their linkage to ecosystem functioning has not yet been entirely revealed [22]. 

The biotic impoverishment underpins the need to understand the role of functional redundancy that might act as the “life-boat” of ecosystem functioning (at least in the short term). Butterflies, a well-studied diverse group of invertebrates of importance for pollination, are sensitive to and reflect environmental changes that are imprinted on ecosystem functioning due to their short life cycle [23,24]. Butterflies are threatened by human-induced disturbances such as land-use intensification and global warming, and are declining worldwide [25,26]. Here, we aim to assess the patterns and drivers of different facets of diversity, and of functional redundancy, using data from the butterfly atlas of Great Britain for the time period of 2005–2009 [27]. We estimated functional redundancy and vulnerability following Mouillot et al. [28] as well as taxonomic and functional richness and β-diversity. We explored the relationship between diversity and redundancy metrics and the effect of temperature, land-use cover, human population and landscape-related variables in shaping the observed patterns of butterfly communities. 

## 2. Materials and Methods

### 2.1. Data and Sampling

We used presence/absence data of butterflies of Great Britain for the time period 2005–2009 [27]. British butterfly data are appropriate for this analysis, as the country is exceptionally well explored [29,30,31,32,33]. The data were collected in accordance with the British and Irish National grid of 10 km × 10 km (100 km^2^). We included in our analyses data for 57 butterfly species. We used only mainland Britain grid cells with more than 50% terrestrial land cover with available environmental data (i.e., 2402 grid cells). Finally, in the beta-diversity analyses, we included 2274 grid cells as the functional β-diversity index requires at least five species to be estimated.

### 2.2. Environmental Data

To explore the effect of climate on butterfly diversity and redundancy, we included in all analyses the mean annual temperature. The mean annual temperature was estimated using the monthly climatic data retrieved from the HadUK-Grid Gridded Climate Observations dataset [34] for the time period 2005–2009, i.e., the years that butterfly distribution was collected, and 2000–2004, i.e., five years before the collection period to take into account time-lag effects of temperature on diversity and redundancy patterns. Additionally, we estimated the percentage of different land use per grid cell. To do so, we obtained gross land cover data from the HILDA maps [35] for the year 2010 (spatial resolution of 1 km^2^). The extracted data included the following land cover classes: (i) forest, (ii) cropland, (iii) grassland, (iv) other (sparsely vegetated areas, beaches and bare soil) and (v) water. Using these data, we estimated the Shannon diversity index of land uses per grid cell to quantify heterogeneity of land uses. The total human population per grid cell was calculated using the Global Human Settlement Layer [36] for the year 2010. Finally, a digital surface model created under the EU GMES/Copernicus program’s reference data access (RDA) action [37] was used to calculate the mean and standard deviation of elevation per grid cell.

### 2.3. Diversity and Redundancy Metrics

We estimated species richness and functional richness per grid cell. To estimate functional richness, we selected four traits that have been linked to responses of butterflies to environmental changes [23,38]: (a) body size quantified by the average wing span of males and females (continuous, mm), (b) voltinism (continuous, number of generations per year), (c) overwintering stage (egg, larvae, pupae, adult) and (d) diet preference (monophagous, broad oligophagous, polyphagous). Trait data were retrieved from the European & Maghreb Butterfly Trait Database [39]. Continuous traits were transformed into categorical traits. Specifically, we used the following categories for (i) body size: 20–30, 31–40, 41–50, 51–60 and >60 and (ii) voltinism: 1, 2, 3, 4 and 5. We estimated Gower species distance and implemented principal coordinates analysis (PCoA) on the distance matrix, and then we estimated the functional richness (FRic) using the dbFD function of the R package FD [40]. Additionally, we estimated the standardized effect size of functional richness using the null model permatswap of the R package vegan [41], which shuffles the species presences within the species by site matrix, keeping the row and column sums constant. We performed 1000 randomizations and SES was estimated by subtracting the mean value of the functional diversity index of the randomized communities from the observed value and dividing by the standard deviation of the value of randomized communities. 

We estimated functional redundancy (FR) and functional vulnerability (FV) per grid cell following Mouillot et al. [28]. The estimation of functional redundancy and functional vulnerability is based on the functional entities defined as the unique combination of functional traits within an assemblage. First, species were assigned to functional entities according to the functional traits used to estimate functional richness, i.e., body size, voltinism, overwintering stage and diet preference. Then, the functional redundancy and functional vulnerability indices were estimated with the following formulae [28]: $Functional\;Redundancy=\frac{\sum_{i=1}^{FE}n_{i}}{FE}=\frac{S}{FE}$ and $Functional\;Vulnerability=\frac{FE-\sum_{i=1}^{FE}min(n_{i}-1,1)}{FE}$, where *FE* is the total number of functional entities, *S* the total number of species in a community and *n_i_* the number of species in a functional entity. By definition, functional redundancy is the mean number of species per functional entity and functional vulnerability is the proportion of FEs with one species. Thus, functional redundancy quantifies the mean number of species sharing identical combinations of functional traits within the community, while functional vulnerability quantifies the mean number of species with unique combinations of functional traits within the community. Functional redundancy takes values between 1, in the case where all functional entities include only one species, and the value of species richness when all the species present in the community belong to one functional entity, i.e., all species have identical functional traits. Functional vulnerability varies between 0, in the case where all functional entities include more than one species, and 1 when all functional entities include only one species. The assignment of species to functional entities and the estimation of functional redundancy and vulnerability were performed using the functions provided in Mouilot et al. [28]. Finally, we estimated the taxonomic and functional pairwise Jaccard dissimilarity index using the betapart R package [42]. 

### 2.4. Statistical Analysis

We explored the relationship of (taxonomic and functional) diversity, functional redundancy and functional vulnerability using generalized additive modeling (GAM) [43]. We explored the relationship of all pairwise combinations of the diversity, redundancy and vulnerability metrics. We assumed a Gaussian error distribution and used penalized thin plate regression splines with k set to 3. Next, we implemented GAMs predicting species richness, functional richness, functional redundancy and functional vulnerability as functions of temperature and land cover, land use heterogeneity, elevation (mean value and variability) and human population (Gaussian error distribution; penalized thin plate regression splines k = 3). Given that environmental variables might by highly correlated, we tested for multi-collinearity issues among predictors by estimating variance inflation factor (VIF) with the function vifstep (with criterion VIF < 10) of the usdm R package [44]. The analysis showed multi-collinearity among variables, and grassland-cover was excluded by any subsequent analyses. We built a separate model for species richness, functional richness, functional redundancy and vulnerability. In all GAMs, we included coordinates of grid cell’s centroid as smooth predictor to account for spatial autocorrelation. Additionally, we built models predicting functional richness, functional redundancy and functional vulnerability as function of the grid cells’ coordinates solely to relatively quantify abiotic factors’ contribution (spatial GAM). The modeling was performed using the mgcv R package [43].

In the next step, we explored the relationship between the taxonomic and functional β-diversity, both quantified by Jaccard pairwise dissimilarity index with GAMs, and included in the model geographical distance between sites to account for spatial autocorrelation. To examine the contribution of environmental variables and geographical distance in shaping taxonomic and functional β-diversity, we performed generalized dissimilarity modeling (GDM) with the package GDM [45]. GDM is a non-linear regression matrix approach that fits I-spline basis functions for each predictor, and the coefficients of the functions are estimated by the maximum-likelihood approach. The coefficients’ sum represents the relative contribution of each predictor in shaping β-diversity (with all other variables constant) and the shape of the curve indicates how the rate of β-diversity changes along the environmental and geographical gradient, allowing us to pinpoint which predictors’ range has a more significant impact on the differences in species and functional composition [45,46]. We used as response variables the taxonomic and functional β-diversity (one GDM for each β-diversity facet) and as predictors (modeled with the three I-spline basis functions per predictor) the mean annual temperature, land-cover uses, the human population, the elevation (mean and standard deviation) and geographical distance. Following this, to disentangle the contribution of environmental variables and geographical distance, we partitioned the deviance explained by GDM into the deviance explained solely by environmental variables, solely by geographical distance and their shared effects. The partitioning was performed with the gdm.partition.deviance of the GDM R package. Finally, to explore the spatial patterns of taxonomic and functional β-diversity, we generated red-green-blue (RGB) color maps. To do so, we applied principal coordinate analysis (PCoA) with the package ape [47] on the predictions of GDM of taxonomic and functional β-diversity and visualized the first three ordination axes with red, green and blue color scales, and their combination, into one map. In these maps, wider differences in species and functional composition are highlighted by greater differences in color. 

All analyses were performed with the R version 4.3.0, R Foundation for Statistical Computing, Vienna, Austria [48].

## 3. Results

Temperature followed the latitudinal gradient, with lower temperatures observed in the northern part of the island, while there was no clear longitudinal gradient (Supplementary Material, Figure S1a). Croplands dominated in the south-eastern part (Supplementary Material, Figure S1b), forest cover was higher but patchily distributed in northern Great Britain, i.e., in the areas of higher elevation or exhibiting elevation variability (Supplementary Material, Figure S1c,j,k), while grassland cover was higher in the north and south-west (Supplementary Material, Figure S1d). Other land uses (sparsely vegetated areas, beaches and bare soil) were detected in the higher elevation areas of northern Great Britain (Supplementary Material, Figure S1e). Settlements were patchily distributed (Supplementary Material, Figure S1f), while water was uniformly distributed (Supplementary Material, Figure S1g). Heterogeneity of land uses was primarily lower in the central part of the island (Supplementary Material, Figure S1h). 

**Figure 1:**
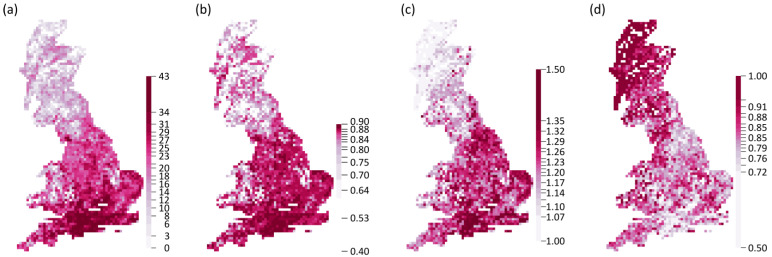
The spatial patterns of taxonomic diversity quantified by (**a**) species richness, (**b**) functional diversity quantified by functional richness, (**c**) functional redundancy and (**d**) vulnerability of British butterflies for the time period 2005–2009.

We detected a relatively strong longitudinal and latitudinal gradient in species and functional richness and metrics of functional redundancy and vulnerability (Figure 1). The basic relationships of species richness have been previously reported [31,32]. Specifically, higher values of species (mean value = 20.52 ± 8.51) and functional (mean value = 0.77 ± 0.16) richness, were observed in the southern and eastern part of Great Britain where the communities were more functionally redundant (mean value = 1.19 ± 0.11). On the other hand, communities were more vulnerable in the northern part (mean value = 0.84 ± 0.08). 

**Figure 2:**
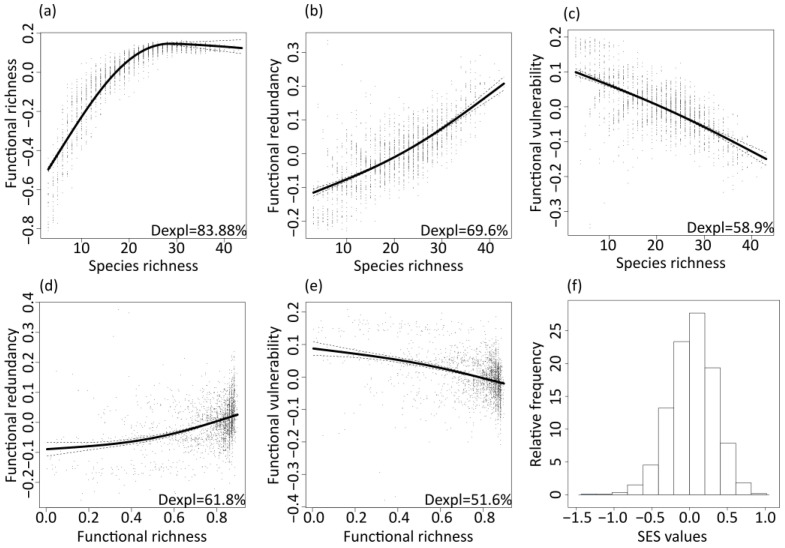
The relationships between species richness and functional metrics: (**a**) richness, (**b**) redundancy, (**c**) vulnerability, and between functional richness and (**d**) functional redundancy and (**e**) functional vulnerability, after accounting for spatial autocorrelation, along with the deviance explained and the partial residuals (points) of the formulated generalized additive models, along with (**f**) the histogram of mean standardized effect size of functional richness of butterflies of Great Britain in the time period 2005–2009.

There was strong evidence, taking into account spatial autocorrelation, that functional richness was related to species richness (*p* < 0.001, deviance explained = 83.88%). Specifically, functional richness increased with species richness up to approximately 27 species and then reached a plateau (Figure 2a). The deviance explained of GAM with predictors being only the coordinates of grid cells (spatial GAM) was equal to 35.20%. Functional redundancy tended to increase with both diversity aspects (species richness: *p* < 0.001, deviance explained 69.60%, Figure 2b; functional richness: *p* < 0.001, deviance explained 61.80%, Figure 2d). The spatial GAM for functional redundancy explained the 57.70% of functional redundancy’s deviance. Thus, in the case of the functional redundancy—functional richness relationship, given also the scattered relationship, the deviance explained was mainly due to the contribution of spatial variables. The functional vulnerability decreased with species richness (*p* < 0.001, deviance explained = 58.9%, Figure 2c) and functional richness (*p* < 0.001, deviance explained = 51.60%, Figure 2e). In this case, spatial GAM exhibited deviance explained equal to 47.10%. Regarding the SES of functional richness, the majority of SES values were equal or close to zero; thus, observed functional diversity does not deviate from random expectations, given the species richness (Figure 2f). 

**Figure 3:**
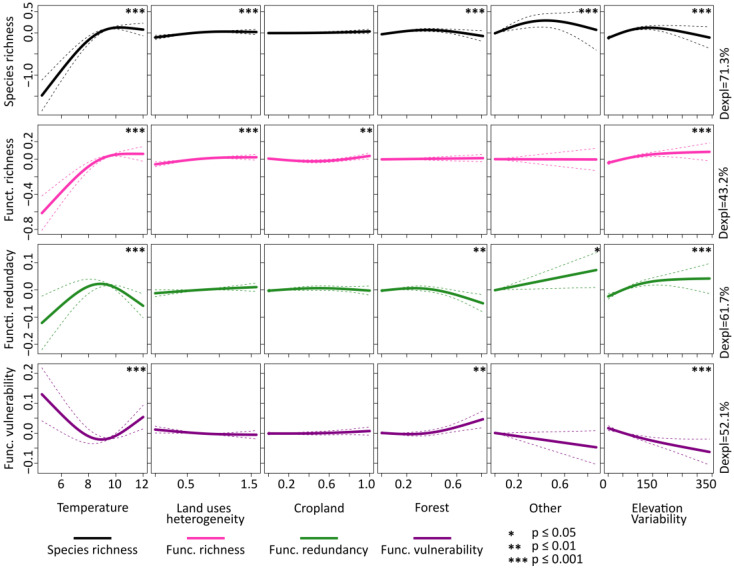
Partial residual plots showing the results of the generalized additive models (shape and significance) predicting taxonomic diversity (species richness), functional diversity (functional richness) and redundancy metrics (functional redundancy and functional vulnerability) of butterflies of Great Britain in the period 2005–2009 as function of temperature, land cover and elevation variability. In the figure, the predictors affecting the majority of diversity and redundancy metrics are presented.

The GAMs predicting diversity and redundancy metrics as a function of abiotic variables—including coordinates of grid cells as predictors—exhibited a relatively high explanatory power in all cases. The relationships (shape and strength) between diversity, functional redundancy and functional vulnerability with predictors that primarily affect the majority of diversity and redundancy are illustrated in Figure 3. Additionally, Supplementary Material Figure S2 presents the relationships’ form and strength with all predictors. Temperature exerted a significant and strong effect on all metrics. Taxonomic richness and functional richness increased with temperatures up to 9 °C and then reached a rough plateau. Functional redundancy had a unimodal relationship with temperature, and vulnerability showed the inverse relationship with temperature. Additionally, both diversity facets increased with heterogeneity of land uses. Species richness had a unimodal relationship with forest cover, other uses cover, settlements cover and elevation variability, while it increased with water cover. Functional richness had a U-shaped relationship with cropland, and increased (non-linearly) with elevation variability. Functional redundancy increased with other uses cover (linearly) and elevation variability (non-linearly), and decreased with forest cover—beyond a threshold equal approximately to 0.4—as well as with elevation variability. Finally, vulnerability increased with forest cover and decreased with elevation variability.

**Figure 4:**
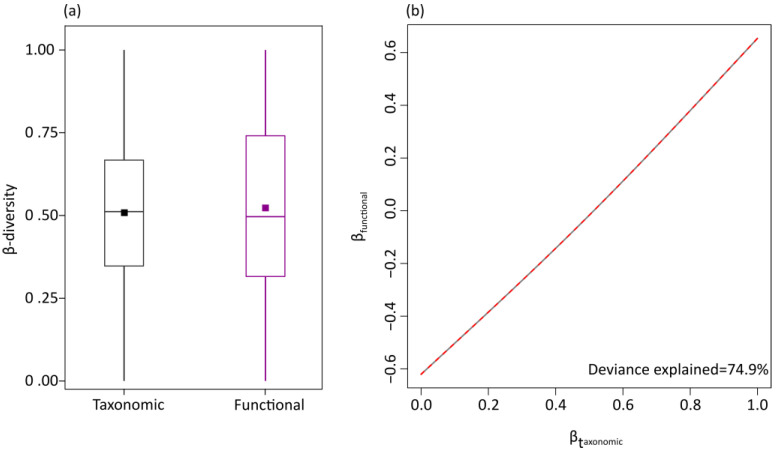
(**a**) Boxplot showing the median values (solid line), inter-quartile ranges (25–75%, box) and mean values (square symbols) of taxonomic and functional β-diversity (**b**) and the relationship between taxonomic and functional β-diversity according to the results of the generalized additive models predicting functional β-diversity as a function of taxonomic β-diversity of butterflies of Great Britain in the period 2005–2009 (red line).

**Figure 5:**
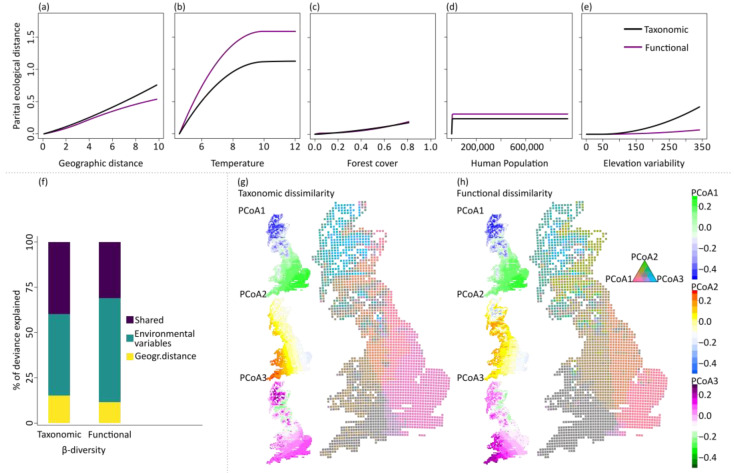
Summary plot showing the results of the generalized dissimilarity models (GDMs) of taxonomic and functional composition of British butterflies for the time period 2005–2009: (**a**–**e**) the fitted I-splines of the GDMs, (**f**) the percentage of deviance explained from geographical distance, environmental variables (including temperature, land cover, human population and elevation (mean value and variability) and their shared effects and spatial patterns of (**g**) taxonomic and (**h**) functional dissimilarity as derived from GDM predictions.

Taxonomic and functional β-diversity, quantified by the Jaccard pairwise dissimilarity index, exhibited similar mean values (Figure 4a). The two facets were highly congruent (Figure 4b). The generalized dissimilarity model explained approximately 49% and 24% of β_taxonomic_ and β_functional_, respectively. Thus, variation in species composition was more strongly driven by abiotic factors than variation in functional composition, although the two diversity aspects respond similarly to the examined abiotic factors (Figure 5a–e). The most important drivers of taxonomic and functional β-diversity were temperature and geographical distance (Table 1). The taxonomic and functional dissimilarity increased linearly with geographical distance (Figure 5a), while they increased with temperatures up to about 9 °C and then reached a plateau (Figure 5b). Additionally, forest cover promoted taxonomic and functional dissimilarity (Figure 5c). Functional dissimilarity increased steeply with human population in sparsely populated areas (Figure 5d), and taxonomic dissimilarity increased with moderate to higher levels of elevation variability (Figure 5e). The unique effects of the environmental variables explained the bulk of variation in species and functional composition (Figure 5f). The predicted spatial patterns of taxonomic and functional β-diversity showed differentiations of composition along the longitudinal and latitudinal gradient (Figure 5g,h). Furthermore, differences in species composition were related to the environmental gradient, primarily to temperature and secondarily to land cover (Figure 5g,h). 

## 4. Discussion

Our results showed that functional richness increased with species richness, but approached a plateau in the most species-rich communities. This saturating relationship suggests a degree of overlap in the functional roles of species, i.e., a higher level of functional redundancy in the more diverse communities. According to Petchey and Gaston [49], the relationship between species richness and functional richness depends on the number of used traits included in the estimation of functional diversity, with the relationship transforming from saturating to positive when the number of traits used increases. Thus, it remains an open research question whether analyzing more traits or traits with more detailed classification schemes would lead to lower levels of functional redundancy. The shape of the relationship between species richness and functional richness depends on the spatial scale and on environmental heterogeneity (for a review, see [50]), while topological complexity might favor butterfly species richness. At large spatial scales and across taxa and regions, studies showed that the relationship can be positive [51,52,53], negative [52] or saturated [54,55,56]. 

Functional redundancy increased approximately linearly with species richness for butterflies of Great Britain, with species-richer communities being less functionally vulnerable. The functional richness-functional redundancy and functional richness-functional vulnerability relationships were similar with the ones observed in the case of species richness, but weaker. The increase in functional redundancy with species richness, as the one observed here, has been reported for other taxa [57,58,59]. Such a positive relationship suggests that the loss of species can impact functional composition of species-poorer communities. Ecosystem functioning is often assumed to rely mostly on common species (widely distributed and abundant species), and not on rare species (narrowly distributed and of low abundance) that are more prone to extinction [60]. Thus, one could speculate that the loss of rare species would not affect ecosystem functioning. However, recent studies demonstrate that the contribution of rare species to functional diversity through their unique combinations of traits, and to ecosystem functioning and services, is of immense significance [28,59,61,62,63].

Temperature was a significant predictor of (taxonomic and functional) richness and redundancy metrics. Temperature promoted species richness and functional richness up to approximately 9 °C and then the relationships reached a plateau. Butterflies as ectotherms depend on temperature to ensure normal activity [64]. Temperature strongly influences butterfly diversity, both directly by affecting the physiology of every stage in their life cycle and indirectly by influencing resource availability [64,65]. However, the association between temperature and redundancy was different; it was unimodal. Thus, warm areas may have species-rich butterfly assemblages, but these assemblages appear to have lower functional redundancy than cooler areas of similar richness. Furthermore, communities of warmer regions seem to be more vulnerable. Although butterflies are anticipated to benefit from the climate warming, especially in temperate regions such as Great Britain, it seems that the positive climate warming effect is counterbalanced by the negative effect of habitat loss and fragmentation [66]. Therefore, a possible explanation of the saturating relationships of (taxonomic and functional) richness with temperature, accompanied by a decrease in functional redundancy, could be attributed not to a temperature effect per se, but to the linkage between temperature gradient and land uses distribution. The warmer southern parts of Great Britain experienced greater land use conversion, habitat deterioration and habitat fragmentation [67] which have resulted in a loss of suitable habitats for some butterfly species and the prevailing of species with specific traits (e.g., generalists) [26]. In England, 47% of semi-natural grasslands were lost in the period of 1960–2013, with the majority of these areas converted to arable land or improved grasslands [68] and this loss resulted in abundance decline and range contraction of specialized butterflies [69]. But it is not only the specialized butterflies that are impacted by habitat loss and fragmentation, as distance between suitable habitats has surpassed the dispersal capacity of moderate generalist (regarding resource utilization) butterfly species [70]. For example, parts of south-eastern Great Britain were dominated by croplands and exhibited low heterogeneity of land uses, and also lower-level functional redundancy than expected by the observed species richness. 

Taxonomic and functional richness did not decline in warmer areas, but we observed higher vulnerability in these regions. In other words, we detected that more unique combinations of functional traits were represented by one species, resulting in lower levels of functional redundancy. Therefore, generalists might have prevailed, but specialized functional groups were still present, but represented by fewer species. This finding implies that land-use changes in warmer areas may have a disproportional impact on community functional composition. Taking into account that in warmer areas such as the Mediterranean region, land-use changes, climate change and its effect on water availability act synergistically and negatively affect butterfly diversity, one fears for the future of butterflies and the implications on ecosystem functioning. However, within this fast-changing world, butterflies have adapted to survive, e.g., exhibited shifts in their phenology and thermal adaptations as a response to environmental change [71,72,73,74]. Butterflies have already shifted their range margin towards northern regions as a response to climate change and changes in land uses, even in this temperate region [75]. Phenological shifts and northwards range shifts hopefully might lead to an increase in the species richness of British butterflies, rendering the communities functionally more redundant and thus less vulnerable. Additionally, a potential colonization, triggered by climate change, of butterfly species from continental Europe could further safeguard butterfly communities. However, one should bear in mind that no successful colonization has been reported for a long time period [32] as well as the lag response of species richness of British butterflies to climate change [76].

Environmental heterogeneity, quantified either by land-use heterogeneity or elevation variability, emerged as an important predictor of diversity and redundancy metrics. The positive effect of land use heterogeneity has been previously reported for butterfly diversity in other regions [77], and also for the stability of butterfly populations in Great Britain [78]. At coarse spatial scales, greater environmental heterogeneity, perhaps due to an increase in available niche space and the diminishing strength of competition, allows the coexistence of more species that are also more functionally dissimilar [50,79]. Our results confirmed that heterogeneity promotes functional redundancy, rendering the communities less vulnerable. 

The percentage of different land-uses was also a significant predictor of diversity and redundancy metrics. Species richness initially increased with forest cover, but decreased in areas with high forest cover. Given the positive relationship between species richness and functional redundancy and the fact that in most communities functional diversity did not deviate from random expectations, not surprisingly, functional redundancy also decreased with forest cover. The majority of British butterflies prefer open habitats such as grasslands [80]. Areas with higher forest cover are also areas of higher elevation, while forests are related to the presence of edges and clearings, and these conditions might favor specific species with certain functional traits. Finally, land use intensification which poses a major threat on diversity and functional redundancy [81,82] might not be adequately quantified by the HILDA land cover dataset.

Taxonomic and functional β-diversity patterns were highly congruent, indicating that communities with different species compositions tend to also have functionally dissimilar species, as has been previously reported for butterflies [83,84] and for other taxa [85,86]. This congruency might suggest that taxonomic and functional composition might be driven to some degree by similar processes. The two facets of diversity responded similarly to the environmental variables and geographical distance. However, the functional β-diversity was more weakly associated with environmental variables and geographic distance, as was indicated by the performance of the generalized dissimilarity model. Perhaps functional β-diversity is driven more strongly by stochasticity, as has been reported for British birds [87,88], or by other factors that were not taken into account in this study. Temperature was the strongest driver of both taxonomic and functional β-diversity, with unique effects of environmental variables explaining the most significant variation of both facets of β-diversity. Temperature is a major driver of butterfly taxonomic and functional β-diversity as it significantly affects their physiology [83,84,89]. Additionally, processes such as dispersal limitation and evolutionary history seem to play a role, albeit weaker than environmental filtering, in shaping butterfly communities as was indicated by the relative importance of geographical distance and its unique effects. Therefore, British butterfly taxonomic and functional diversity result from a combination of deterministic and stochastic processes, as very often observed in other taxa [90,91,92].

## Figures and Tables

**Table 1:** Summary of the generalized dissimilarity model of species composition and functional composition of butterflies of Great Britain in the time period 2005–2009. The relative importance of predictor variables was assessed by summing the coefficients of the three I-splines of the generalized dissimilarity models.

	Taxonomic β-Diversity	Functional β-Diversity
Deviance Explained	49.13%	25.40%
Variable Importance		
Geographic Distance	0.76	0.54
Temperature	1.13	1.59
Cropland	0.02	0.01
Forest	0.18	0.20
Other	0.01	0.18
Settlement	0.01	0.02
Water	0.00	0.02
Human Population	0.23	0.31
Elevation	0.00	0.00
Elevation Variability	0.43	0.07

## Data Availability

The biotic data are available from Butterfly Distributions for Great Britain for the period 2005–2009 from the Butterfly Conservation and the Biological Records Centre http://data.nbn.org.uk (accessed on 12 December 2012) and https://butterflytraits.github.io/European-Butterfly-Traits/index.html (accessed on 12 December 2012). Climatic data are available at https://catalogue.ceda.ac.uk/uuid/4dc8450d889a491ebb20e724debe2dfb (accessed on 12 December 2012), land use data are available at https://www.wur.nl/en/research-results/chair-groups/environmental-sciences/laboratory-of-geo-information-science-and-remote-sensing/models/hilda/hilda-data-downloads.htm (accessed on 12 December 2012), human population data are available at https://ghsl.jrc.ec.europa.eu/download.php?ds=pop (accessed on 12 December 2012), and elevation data are available at https://land.copernicus.eu/imagery-in-situ/eu-dem/eu-dem-v1.1 (accessed on 12 December 2012).

## Supplementary Materials

The following supporting information can be downloaded at: https://www.mdpi.com/article/10.3390/insects14090722/s1, Figure S1: The spatial distribution of temperature, land-uses cover, human population and elevation (mean values and variability) of Great Britain for the time period 2005–2009; Figure S2: The relationships (shape and strength) between diversity, functional redundancy and functional vulnerability with different environmental factors (temperature, land use heterogeneity, land uses, human populations and elevation).
