# Supplementary material for: How Biodiversity, Climate and Landscape Drive Functional Redundancy of British Butterflies
Source: Insects. 2023 Aug 23;14(9):722. doi: 10.3390/insects14090722 (PMC10531656; doi:10.3390/insects14090722)
Supplement: Supplementary file 1 [file insects-14-00722-s001.zip › insects-2558941-supplementary.pdf]

Supplementary Material

How Biodiversity, Climate and Landscape Drive Functional Redundancy of British Butterflies

Maria Lazarina *, Danai-Eleni Michailidou, Mariana Tsianou, and Athanasios S. Kallimanis

Department of Ecology, Aristotle University of Thessaloniki, 54124 Thessaloniki, Greece; dmichail@bio.auth.gr (D.-E.M.); kalliman@bio.auth.gr (A.S.K.)

* Correspondence: mlazarin@bio.auth.gr

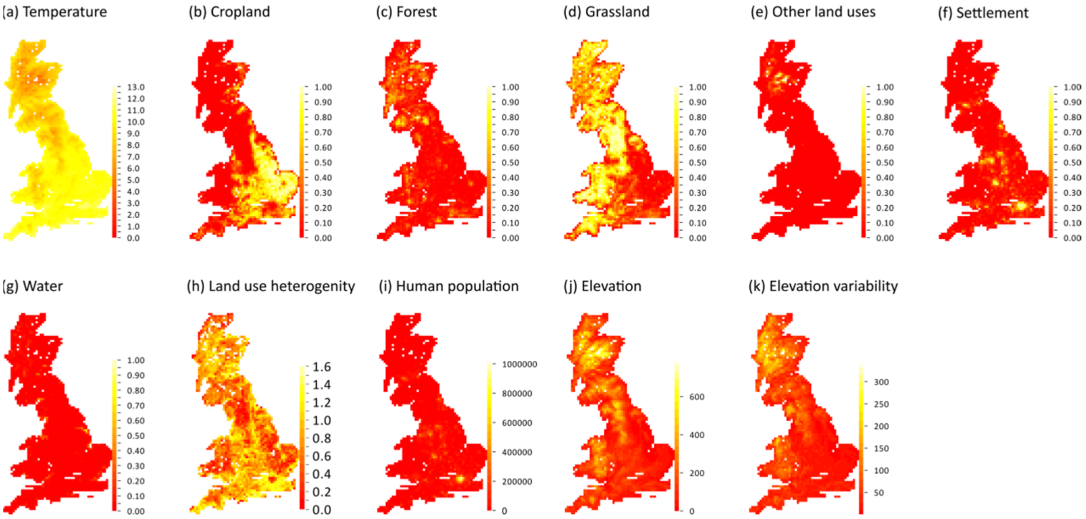

Figure S1. The spatial distribution of temperature, land-uses cover, human population and elevation (mean values and variability) of Great Britain for the time period 2005-2009.

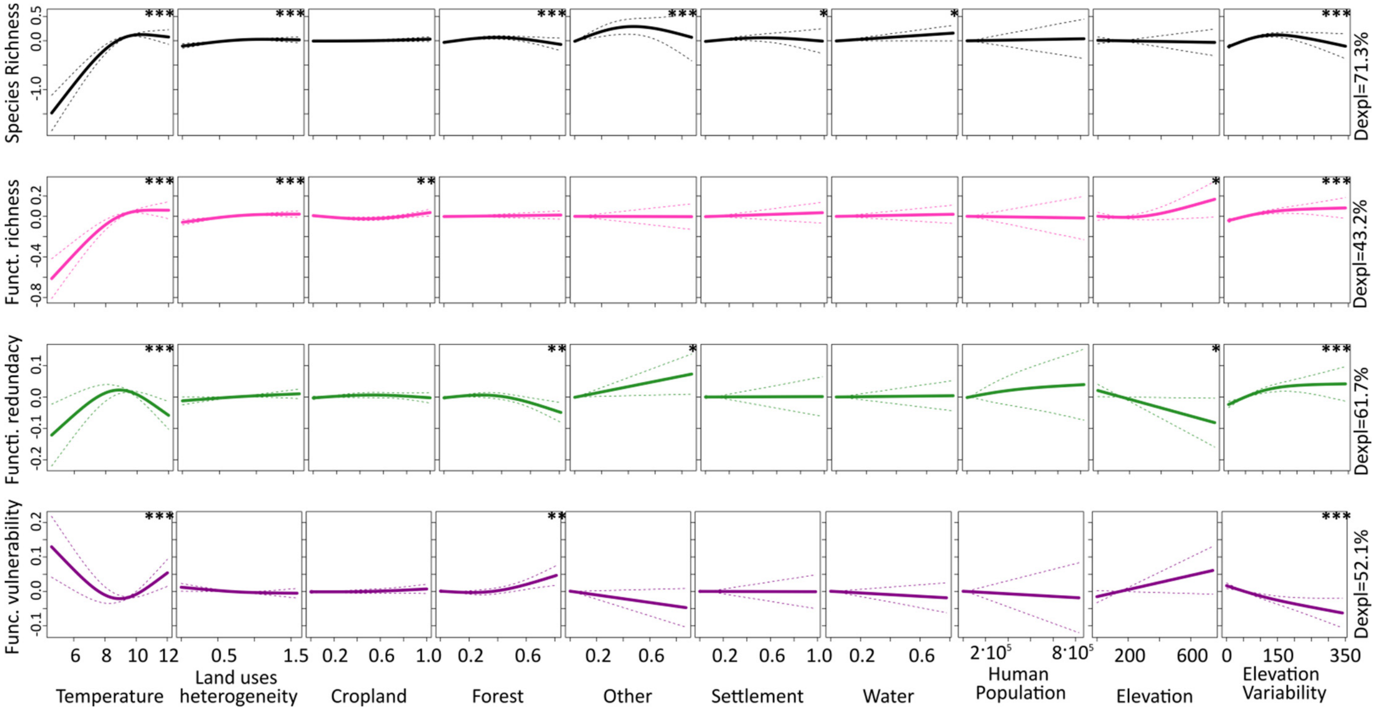

Figure S2. Partial residual plots showing the results of the Generalized Additive Models (shape and significance) predicting taxonomic diversity (species richness), functional diversity (functional richness), and redundancy metrics (functional redundancy and functional vulnerability) of butterflies of Great Britain in the period 2005-2009 as function of temperature, land cover, and elevation variability.
